# Implications of the Immune Polymorphisms of the Host and the Genetic Variability of SARS-CoV-2 in the Development of COVID-19

**DOI:** 10.3390/v14010094

**Published:** 2022-01-06

**Authors:** Jesús Zepeda-Cervantes, Daniel Martínez-Flores, Josué Orlando Ramírez-Jarquín, Ángeles C. Tecalco-Cruz, Noé Santiago Alavez-Pérez, Luis Vaca, Rosa Elena Sarmiento-Silva

**Affiliations:** 1 Departamento de Microbiología e Inmunología, Facultad de Medicina Veterinaria y Zootecnia, Universidad Nacional Autónoma de México, Mexico City 04510, Mexico; mvzchuy@gmail.com; 2 Departamento de Biología Celular y del Desarrollo, Instituto de Fisiología Celular, Universidad Nacional Autónoma de México, Mexico City 04510, Mexico; danielciencias14@gmail.com; 3 Departamento de Neuropatología Molecular, Instituto de Fisiología Celular, Universidad Nacional Autónoma de México, Mexico City 04510, Mexico; jjarquin@ifc.unam.mx; 4 Posgrado en Ciencias Genómicas, Universidad Autónoma de la Ciudad de México (UACM), Mexico City 06720, Mexico; angeles.tecalco@uacm.edu.mx; 5 Escuela Nacional de Medicina y Homeopatía, Instituto Politécnico Nacional, Mexico City 07340, Mexico; noe.alavez@gmail.com

**Keywords:** immune polymorphism, SARS-CoV-2 variant, ACE2 overexpression, genetic susceptibility, severe COVID-19

## Abstract

The severe acute respiratory syndrome coronavirus-2 (SARS-CoV-2) is responsible for the current pandemic affecting almost all countries in the world. SARS-CoV-2 is the agent responsible for coronavirus disease 19 (COVID-19), which has claimed millions of lives around the world. In most patients, SARS-CoV-2 infection does not cause clinical signs. However, some infected people develop symptoms, which include loss of smell or taste, fever, dry cough, headache, severe pneumonia, as well as coagulation disorders. The aim of this work is to report genetic factors of SARS-CoV-2 and host-associated to severe COVID-19, placing special emphasis on the viral entry and molecules of the immune system involved with viral infection. Besides this, we analyze SARS-CoV-2 variants and their structural characteristics related to the binding to polymorphic angiotensin-converting enzyme type 2 (ACE2). Additionally, we also review other polymorphisms as well as some epigenetic factors involved in the immunopathogenesis of COVID-19. These factors and viral variability could explain the increment of infection rate and/or in the development of severe COVID-19.

## 1. Introduction

**Figure 1:**
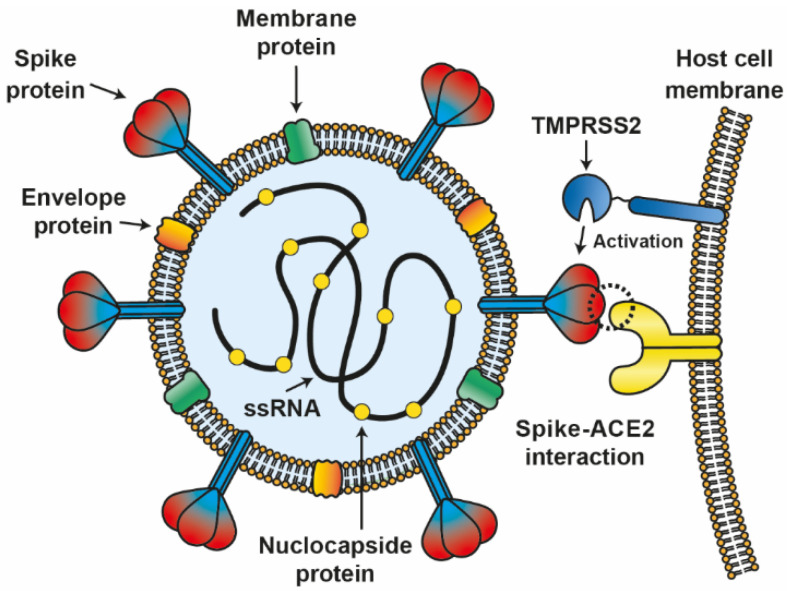
Interaction between SARS-CoV-2 and host cell. The SARS-CoV-2 virion is composed of 4 structural proteins, spike protein (S), membrane protein (M), envelope protein (E), and nucleocapsid protein (N), associated with single-stranded RNA (ssRNA) of the virion. S protein interacts with the host cell ACE2 protein and is activated by TMPRSS2 as part of the infection mechanism.

Coronaviruses have been shown to be a potential agent for pandemics [1]; particularly, coronaviruses from bat origin have been identified with zoonotic potential [2]. Coronavirus disease 2019 (COVID-19) is caused by the severe acute respiratory syndrome coronavirus-2 (SARS-CoV-2). SARS-CoV-2 is a single-stranded RNA enveloped virus that belongs to the *Coronaviridae* family [3,4]. Initially, this virus was isolated and sequenced in China from bronchoalveolar lavage fluid of patients with dyspnea, headache, dry cough, fever, and pneumonia. Genetic analyses revealed that this new coronavirus shares nearly 80% sequence identity with SARS-CoV-1 [2]. SARS-CoV-2 genome is about 30 kilobases, encoding for structural proteins, such as spike (S), membrane (M), envelope (E), and nucleocapsid (N) proteins as well as non-structural proteins (NSPs) [5] (Figure 1).

SARS-CoV-2 can evade the immune system using its NSPs, mainly affecting people with co-morbidities and/or with some type of immunosuppression. NSP1-16 from SARS-CoV-2 are involved in the assembly of viral particles and the viral RNA polymerase [6]. It has been shown that NSP1 from SARS-CoV-1 is an immunosuppressive molecule that inhibits phosphorylation by signal transducer and activator of transcription 1 (STAT1) and hence blocks the interferon (IFN) signaling. Similarly, NSP7 and NSP15 have antagonistic effects on IFN [7]. In addition, mutations in NSP2 and NSP3 have been associated with an increase in the infection capacity of new SARS-CoV-2 variants [6]. Therefore, the IFN response is antagonized by SARS-CoV-2 NSPs, decreasing its levels and contributing to severe infection.

Host factors are also important for the development of severe COVID-19. It is relevant to mention that some people develop severe COVID-19, while others remain without symptoms of this disease. Finding differences between asymptomatic and seriously ill patients due to COVID-19 contributes to a broader knowledge of this disease and could help in the development of new therapies. SARS-CoV-2 binds to the angiotensin-converting enzyme type 2 (ACE2) receptor with high affinity through its glycoprotein S (similarly to SARS-CoV-1) [2,8] (Figure 1). ACE2 mRNA is present in all organs [9]. For viral entry, glycoprotein S from SARS-CoV-2 passes from a pre-fusion to a fusion state by directed cleavage from cathepsin B and L from the endosomal compartment as well as by transmembrane protease serine 2 (TMPRSS2). Primarily, TMPRSS2 plays a critical role in pathogenesis and viral spread, and it has been shown that TMPRSS2 inhibitors block the virus entry [10]. Thus, some host genetic factors associated with the development of severe COVID-19 include the polymorphism of *ACE2, TMPRSS2*, and other transmembrane proteins. ACE2 polymorphisms have been associated with susceptibility to SARS-CoV-2 infection due to its close interaction with its glycoprotein S. Furthermore, ACE2 is overexpressed under inflammatory conditions, such as those produced during SARS-CoV-2 infection [11,12], and is expressed differently among populations, and this could also explain, in part, differences in the severity of COVID-19 [13].

Other examples of host genetic factors associated with severe COVID-19 include immune polymorphisms that have been identified in genes encoding for cytokine, chemokines, their receptors, pattern recognition receptors (PRRs), proteins involved in their signaling pathway, and major histocompatibility complex (MHC), including both MHCI and MHCII. As some examples of PRRs, we can mention polymorphisms of Toll-like receptor (TLR)-3 or molecules involved in their signaling pathways [14,15,16] as well as polymorphisms of RIG-1 and MDA5, which are associated with susceptibility to SARS-CoV-2 infection due to lack for recognition of viral RNA and disrupted signaling pathway for IFN production. In this sense, IFN polymorphisms or polymorphisms of the molecules in charge of signaling for their production, such as IRF3, IRF7, TICAM1, TBK1 [17], and STAT2 [18], have also been related to poor prognosis of COVID-19 patients. It is important to remember that polymorphisms may include changes in the promoter regions or nucleotide coding sequences, including loss of function and missense mutations [17,19]. Thus, the infection rate of SARS-CoV-2 variants and immune system polymorphisms are the main players in the pathophysiology of severe COVID-19.

In the present work, we review information about the genetic susceptibility to SARS-CoV-2 infection based on immune polymorphisms and the implications of SARS-CoV-2 variants in the development of severe COVID-19.

## 2. Genetic Variability of SARS-CoV-2: Implications on Antigenic Drift

SARS-CoV-2 mutants have been isolated from animals, with mammals being the most important, while reptiles, birds, and fishes are less prone to the infection by this virus [20]. Overall, the mechanisms involved in the development of mutations may include (1) an error in the copy process of viral replication; (2) RNA editing by the host, and (3) recombination of two different viral lineages [4,21,22].

Several environmental factors may also induce mutations (UV radiation, metals, or substances produced by organisms). Many mutations have occurred in several regions of SARS-CoV-2 genome, and natural selection favors more infecting viruses. Overall, RNA viruses mutate faster than DNA viruses and with the highest rate [21,23]. In consequence, SARS-CoV-2 has a high probability to mutate, and this makes it most adaptive to the environment.

The effects of mutations are diverse; they can be neutral or synonymous or non-synonymous, affecting the viral processes associated with infection [22]. Besides this, the high error rate of RNA viruses leads to the generation of “quasispecies” or mutants. The resultant diversity of variants may prompt adaptation or viral extinction [22,24]. Additionally, coronaviruses use non-structural proteins (such as NSP3 and NSP4) that form products within vesicles and/or attached to the membrane that the innate immune system receptors cannot detect during replication. Moreover, N protein packages the viral RNA at the end to protect it from degradation. In general, respiratory viruses also form membranous networks to prevent the immune system from recognizing their nucleic acids [25].

### 2.1. Mutations in S Glycoprotein

The emergence of new SARS-CoV-2 variants resulting from the accumulation of mutations could delay the end of the COVID-19 pandemic. Emergent variants are monitored by the Global Initiative on Sharing All Influenza Data [26]. A classification has been proposed, and three variant types have been defined: variants of interest (VOI), variants of concern (VOC), and variants of high consequence [27]. This latest classification of SARS-CoV-2 variants is based on S mutations and includes variants: alpha, beta, gamma, delta, and omicron, some of which have unfortunately increased their infectivity ratio and/or reduced antibody neutralization capacity.

**Figure 2:**
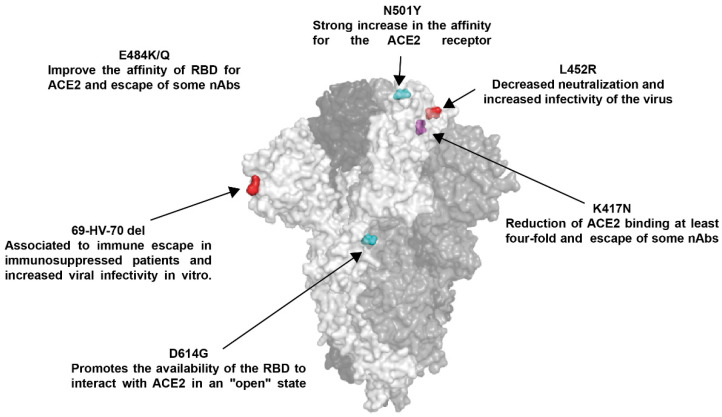
Main mutations in S glycoprotein that regulate viral transmissibility and neutralization. Structure of the S glycoprotein in a trimeric conformation (PDB: 6VXX); each monomer is shown in a different shade of gray, mutation sites are only shown for one monomer (light grey), and the colors used are to visualize one mutation for another.

**Figure 3:**
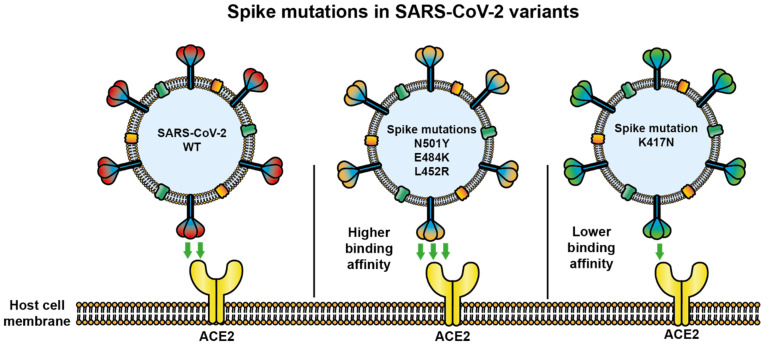
SARS-CoV-2 variants modify their binding capability to angiotensin converting enzyme 2 (ACE2). Variants of SARS-CoV-2 with altered glycoprotein S can modify their interaction with the viral receptor ACE2 by increasing its binding capacity (variant N501Y, E484K, or L452R) or inhibiting it (variant K417N) during infection.

Currently, four VOC are reported: B.1.1.7 was first reported in the United Kingdom (UK) (alpha), followed by B.1.351 of South Africa (beta), B.1.617.2 of India (delta), and P.1 of Brazil (gamma) [27], in which S mutations have received special attention (Table 1, Figure 2 and Figure 3) since mutations can result in improved host receptor affinity [28], improved suitability of viral infection [28,29], reduced efficacy of treatments [30], potential diagnostic impact [30], and reduced neutralization by antibodies generated against previous infection or even vaccination [31].

The first S mutation of impact emerged in late February, consisting of the substitution of Asp 614 by Gly (D614G), G614 quickly replaced D614, as the dominant pandemic form, associated with improved viral infectivity [32]. G614 mutation in S glycoprotein promotes the availability of the receptor binding domain (RBD) to interact with ACE2 in an “open” state [29], contributing to the entry of the virus and eventually in its transmission [33]; in addition, it has been associated to resistance to proteolytic cleavage during the production of the protein in the host cells [34]. Fortunately, G614 does not appear to interfere with the protection provided by natural infection [35].

Currently, G614 is present in all VOC along with other S mutations, such as those present in RBD [36,37]. In B.1.1.7 variant, the G614 mutation is accompanied by N501Y in the RBD, and this mutation has been associated with a strong increase in the affinity for the ACE2 receptor [28,38,39], which may explain a high viral load and faster spread, causing high transmissibility, so far not associated with higher mortality [3,40].

In addition to the D614G and N501Y mutation in RBD, variants B.1.351 and P.1 have acquired the E484K/T mutation in the RBD, respectively [41]. Variants with the K484 mutation have been reported to improve the affinity of RBD for ACE2 but only 1.4-fold [28]. They have also been related to the evasion of the immune response [42,43,44]. On the other hand, the K417N mutation is also present in variants B.1.351 and P.1, and this results in a reduction of ACE2 binding by at least four-fold [28]; however, it has been identified as a determinant for the evasion of the induced immune response in previously infected individuals [43,44,45]. The recent emergence of variant B.1.617.2 has shown that other S glycoprotein mutations should be monitored; this variant contains the mutation L452R, associated with an improvement in viral infectivity due to a higher affinity of RBD for ACE2 [46,47,48].

Multiple S mutations can favor SARS-CoV-2 infection capacity, the evasion of the immune response, improved expression, and resistance to treatments and even diagnosis, properties that may have an additive effect for some variants; therefore, vigilance regarding their progression should be continuous.

### 2.2. Mutations in Other SARS-CoV-2 Proteins

In addition to S glycoprotein, SARS-CoV-2 contains several proteins, including N protein and RNA-dependent RNA polymerase (RdRp/NSP12) as well as NSP1-16, L-, and S-type proteins [49]. This virus’s whole genome is composed of the following 11 genes: *open reading frame* (ORF) *1ab*, *ORF1a*, *ORF2*, *ORF3*, *ORF4*, *ORF5*, *ORF6*, *ORF7*, *ORF8*, *ORF9*, and *ORF10*. From these ORFs, *ORF1* and *ORF2* are the biggest in the genome. *ORF1* is comprised of two ORFs: *ORFa1* (which includes NSP1–NSP11) and *ORF1ab* (which includes NSP12–NSP16). The second largest protein is the S glycoprotein (gene *S*, encoded in *ORF2*) [23,50]. Although the mutations on S glycoprotein receive significant attention, there are many other mutations identified in non-S genes of SARS-CoV-2. Among these mutations, there have been identified some synonymous mutations in the *ORF1a* region. These mutations were found in the third nucleotide for each codon [51]. However, in *NSP6*, a mutation was reported that modified its amino acid sequence. The mutation G11083T modified TTG (leucine) to TTT (phenylalanine). The hydrophobicity profile is equal for both amino acids; however, the side chain is different, which may produce NSP6 to fold differently [50,52,53,54,55,56]. NSP6 is required to produce autophagosomes, the organelles needed for intracellularly degradation via lysosomal. These autophagosomes are involved in the virulence of SARS-CoV-2 [57,58].

In the *ORF1ab*, which contains NSP12A, NSP13, and NSP14, four mutations have been reported. Two of these mutations are synonymous and are located in the proteins NSP12a and NSP13. Another mutation is located in NSP13 protein at position A17858G, which changed ATG (methionine) to GTG (valine). This results in a modification from a polar hydrophilic amino acid to a nonpolar hydrophobic amino acid, resulting in a different folding and functionality of the protein. The fourth mutation is also located in NSP13 at the C18060T position. The codon is modified from TCT (serine) to TTT (phenylalanine), and the effect is the same as A17858G mutation. It has been suggested that the NSP13 protein has helicase activity, suggesting that virus RNA unfolding can be altered by these mutations. Notably, these missense mutations are very similar in their effect, which may indicate that these are not random mutations but probably represent a common mechanism that helps the virus to develop an adaptive response [23].

The second gene, *ORF2*, encodes the Spike glycoprotein, and mutations have been discussed previously and separately [28,38,39]. The *ORF3a* gene contains the mutation G25566T, which changes AGA (arginine) to ATA (isoleucine). The effect of this mutation is to change a highly polar hydrophilic to a non-polar hydrophobic amino acid. This may alter the folding and function of this protein [23]; however, more studies are necessary to clearly determine the effects of these mutations.

One mutation reported for the *ORF8* gene (T28144C) is a synonymous mutation for phenylalanine. This mutation does not modify the folding of the protein; nevertheless, it is suggested that it may help to “cloak” virus in humans or affect transmission from human to human [23]. However, there are not enough reports to support this proposal. In addition, three important mutations have been detected in NSP13: C17747T, A17858G, and C18060T. It is important to note that this protein is highly conserved in some other viruses, and two of these three mutations are not silent mutations, making them highly interesting to carry out studies and determine their functional importance on virus infectivity. The SARS-CoV-2 has a high contagion rate producing a worldwide spread and a serious health problem [23,50,53,59,60,61].

It was recently reported that there are eight mutations in the SARS-CoV-2 genome that modified the amino acid sequence; such mutations are C1059T, G11083T, C14408T, A23403G, G25563T, G28881A, G28882A, and G28883C. The mutation C14408T in NSP12 was the most prevalent. Furthermore, in ORF3a, a frequent mutation was reported: C241T in the 5′UTR, which may modify expression, regulation, and gene assembly [62]. Some other mutations have been identified in other accessory proteins of this virus. In the ORF1a region, C1059T, C3037T, G11083T, and C14408T have been reported; they appear in NSP2, NSP3, NSP6, and NSP12, respectively. The effect of these mutations is not totally understood. The C1059T mutation changes the threonine by isoleucine at position 266 of NSP2 [63]. The G11083T mutation changes leucine by phenylalanine (L36F) of NSP6, and it has been associated with the formation of vesicles involved in microtubule regulation. In the NSP12, the C144048T and C14805T mutations have been detected. The first mutation changes proline to leucine at amino acid 232 (P232L), and it is proposed that it may contribute to virus dissemination. The second mutation has been related to protein replication and to the pathogenicity of SARS-CoV-2.

Among structural protein mutations, some of them have been reported in the protein N. This protein has an important role in regulating the metabolism of infected cells as well as replication and transcription. Three mutations have been identified in this protein: G2881A (arginine to lysine, R204K), G28882A (R204K), and G28883C (glycine to arginine G205R) [64].

To this date, there are several variants of SARS-CoV-2 that have evolved in a country-specific manner. All these mutations may have an important role in providing adaptation mechanisms to the SARS-CoV-2 and increasing spreading among several countries and worldwide. For these reasons, it is important to detect and understand the many mutations in all SARS-CoV-2 proteins and not just protein S mutations (which have been the focus of most studies).

### 2.3. Antigenic Changes in SARS-CoV-2

Mutations are classified as re-assortment, recombination, and antigenic drift. The high mutation rate of some RNA viruses, as occurs with the influenza virus, causes loss of effectiveness of the vaccines and can also generate interspecies jumping promoting zoonoses [65,66]. Antigenic drift and antigenic shift are commonly observed in influenza viruses, whereas with coronaviruses, recombination is often seen [67]. Besides, coronaviruses present a great diversity, and this is due to the low fidelity of RdRp, coupled with the fact that coronaviruses have a long genome that presents a high recombination rate [66]. SARS-CoV-1 and Middle East respiratory syndrome coronavirus (MERS-CoV) have emerged from animal’s coronaviruses, mutating through infection in animals to reach humans [68,69,70,71]. Particularly, in SARS-CoV-2, the variant B.1.1.7 (also known as VOC 202012/01) quickly became predominant due to its high transmission potential [41,72]. Another SARS-CoV-2 variant (from South Africa, variant 501Y.V2) contains the mutations K417N, E484K, and N501Y in the S glycoprotein. This variant probably was originated in an immunocompromised individual with prolonged infection [41,73].

On the other hand, many emerging and re-emerging diseases present recombination. A clear example of this is the fact that a coronavirus was isolated in turkeys, which was a recombinant of the infectious bronchitis virus that infects gallinacea family members, containing in its genome the gene *S* of coronavirus 122 [67]. Thus, recombination among several SARS-CoV-2 variants could occur under suitable conditions favoring the emergence of new strains. The antigenic divergence of SARS-CoV-2 characterized by new glycosylation sites may have contributed to this virus generating the COVID-19 pandemic [74].

Viral protein glycosylation is mediated by host–cell machinery. S protein is a target of glycosylation, resulting in viral peptides masked with glycans from the host to evade the immune system [75]. The virus can be protected from neutralization via N-glycosylation of SARS-CoV-2 RBD [76]. It has been proposed that the glycosylation of S protein may have an impact on the generation of antibodies and vaccines [77]. Furthermore, glycosylation affects the interaction between S glycoprotein and its receptors. In a study, N331 and N343 N-glycosite mutants of SARS-CoV-2 RBD were expressed and purified, demonstrating that de-glycosylation at N331 and N343 decreases virus-receptor interaction. Thereby, viral internalization into respiratory epithelial cells is facilitated by N-glycosylation of SARS-CoV-2 RBD [76]. In another work, 22 N-glycosites and site-specific N-glycans in the S protein were reported. This modification was enriched in the S1 and S2 subunit, responsible for receptor binding and membrane fusion, respectively [78]. It was also demonstrated that S protein is an O-glycoprotein, displaying a variety composition of O-glycosites and O-glycan in a host cell type-dependent manner [79]. Additionally, the size of O-glycan in the glycosylated S protein appears as a factor that modulates the affinity of virus-receptor interaction. An increase in the size of O-glycan in the S protein enhances its interaction with the ACE2 receptor [80]. In addition, it has been suggested that to enter human cells, SARS-CoV2 requires furin protease [81]. A cleavage site for furin was identified in the S1/S2 boundary of SARS-CoV-2 S glycoprotein. This furin cleavage site in S glycoprotein is processed during biosynthesis. Interestingly, a glycan near the S1/S2 boundary can affect the proteolytic activity on S protein [82]. Furin proteolytic activity seems to be modulated by the O-glycosylation of the S protein, influencing viral infectivity and tropism [83]. Thus, glycosylation of S protein is a critical modification that demands attention to develop novel strategies against SARS-CoV-2.

It is well known that several mutations occur in S glycoprotein, the target of SARS-CoV-2 vaccines. A variant of SARS-CoV-2 in a single mutation in S glycoprotein (variant D614G) is more susceptible to neutralizing antibodies than SARS-CoV-2 containing D614; this may be because the S glycoprotein containing D614G has a conformation that can allow the binding of neutralizing antibodies to S epitopes [84]. On the other hand, SARS-CoV-2 variants accumulating important mutations can evade the humoral immune response due to few but important amino acid substitutions, as observed with the delta variant [85].

As we mentioned above, there have been a lot of mutations that modify the biological characteristics of SARS-CoV2, such as antigenicity, transmissibility, and infectivity [36]. D614G (aspartic acid changed to glycine) mutation is one of the major mutations reported that increase the infectivity [29] and transmissibility [86] from SARS-CoV-2. This mutation can prompt a change in the structure of S glycoprotein or modify its epitopes [32,87]. In addition to D614G mutation, it has been reported the modifications N439K and Y453F. N439K substitution increases the binding to ACE2 receptor [39,88], reducing the neutralizing activity of antibodies. Furthermore, N439K substitution has been observed companied by a deletion in amino-terminal domain (∆69–70), and it is probable that these changes increase the infectivity of SARS-CoV2 [89]. These mutations have been reported in the immunodominant region RBD.

Other mutations that have been proposed to help SARS-CoV2 to avoid the immune system are found in E484 (modifying by K, Q, or P, which can reduce neutralization) [90] and S477 sites (changing by G, N, and R), producing resistance to some sera [45].

Several mutations have been identified to affect the response to immune system despite not being localized in RBD. In the amino-terminal domain of S protein, there have been observed some changes that may increase the immune escape. Five deleted regions have been identified in this domain: ∆69–70, ∆141–144, ∆146, ∆21, and ∆243–244 [91]. Other mutations in amino-terminal domain that avoid the immune response include ∆140, N148S, K150T, K150E, K150Q, K150R, and S151P [43,46]. It is relevant to understand and predict the possible relevant sites and/or changes of SARS-CoV-2 that modify its biological properties in order to develop the best immunological strategies to combat this health problem.

## 3. SARS-CoV-2-Host Interaction

### 3.1. Transmission Mechanisms

SARS-CoV-2 enters through mucosal tissues, mainly via oral or respiratory, reaching the intestines and/or alveoli, where it replicates. The viruses located in the airway of infected people reach the upper respiratory tract and conjunctiva of healthy people [92]. At the beginning of infection, SARS-CoV-2 replication occurs in the nasal ciliated epithelium following the lower respiratory tract and pneumocytes [93,94]. Upon SARS-CoV-2 infection, it has a period of incubation that lasts several days, with replication and shedding of SARS-CoV-2 without symptoms [8,95].

Clinical signs of SARS-CoV-2 infection include dry cough, fever, and respiratory distress beginning five days after infection. These clinical signs are present in approximately 98% of symptomatic people. People infected with SARS-CoV-2 sometimes have no clinical signs, but these patients can develop a fulminant disease due to acute respiratory failure and sepsis [96]. Besides, secretory cells release viral particles by exocytosis, and that is why SARS-CoV-2 is found in sputum [97]. This has facilitated the rapid spread of SARS-CoV-2 infection to the population.

Moreover, SARS-CoV-2 and SARS-CoV-1 have similar stability on surfaces, and they can be found in aerosol, where they may stand in the air for hours in insufficiently ventilated areas [98]. In addition, it has been suggested that SARS-CoV-2 could bind to PRRs from dendritic cells (DCs), as it occurs with SARS-CoV-1, and spreads to susceptible cells in the body [99].

### 3.2. Cells Permissive to SARS-CoV-2 Infection

Coronaviruses have a wide host range, although they are isolated mainly from bats, avian species, and less frequently mammals, such as companion animals and livestock [1]. ACE2 is found in all the analyzed human cells, strongly suggesting that SARS-CoV-2 could replicate in almost all types of human cells [9]. The primary target for developing vaccines against SARS-CoV-2 is the S glycoprotein [100]. In addition, SARS-CoV-2 needs TMPRSS2 for viral entry (or cathepsin B and L). Although ACE2 is expressed in all types of organs, it is mainly observed on the surface of epithelia of the medium small-intestine and vascular endothelium [9]. Besides, ACE2 and TMPRSS2 are present in the nasal and bronchial epithelium and in alveolar epithelial type II cells [92]. Because most human cells express the receptor, this virus can infect several cell types and induce different virulence among individuals as well as different clinical symptoms [101]. Additionally, SARS-CoV-2 antigen has been found in the trachea, kidneys, pancreas, small intestine, brain, and blood vessels [102].

### 3.3. SARS-CoV-2 Infection and Immunopathogenesis

The high number of cells permissive to SARS-CoV-2 infection explains the health problems observed during COVID-19, including pneumonia, diarrhea, and hemodynamic problems (associated with deposition of immune complexes in endothelial cells, leading to damage and disseminated intravascular coagulation and subsequently thrombosis). SARS-CoV-2 can also infect the nervous system [103]. Overall, the pathogenesis of COVID-19 is given by an exacerbated response of the immune system. SARS-CoV-2 infection prompts high levels of interleukin (IL)-6, which induces lymphocyte exhaustion [104,105,106]. In addition to this, most COVID-19 patients have a low number of CD4+ and CD8+ T lymphocytes [107]. High levels of pro-inflammatory cytokines by the excessive activation of immune cells constitute the cytokine storm, which is observed in severely ill patients with poor prognosis [104,105,106].

The cytokine storm results in macrophage infiltration and a T-helper 17 (Th17) cell response in the lungs of COVID-19 patients [104]. Here, cytokine storm syndrome constitutes a double-edged sword. Other factors implicated in developing severe disease include complement activation and coagulopathy [108]. Capillary leak and endothelial activation are other characteristics found during hyperinflammation, prompting a circulatory collapse. As a result of capillary disfunction, the clotting cascade is activated, generating thrombosis, leading to multi-organ failure and subsequent shock [109]. Interestingly, it has been suggested that patients with severe COVID-19 contain low levels of IFN and not necessarily high viral titers [110].

On the other hand, older people are more susceptible to SARS-CoV-2 infection. Elderly patients have a lower capacity to respond to viral infections, and their basal levels of pro-inflammatory cytokines are higher than in children [11]. This can be partially explained by the development of immunosenescence, which is characterized by an increase in CD28+ T cells, a low number of T cells in the bloodstream, and chronic inflammation. Additionally, these factors are related to chronic heart failure [107].

## 4. ACE2 Polymorphisms in COVID-19

### 4.1. ACE-2: Global Structure

ACE is a type 1 transmembrane glycoprotein, specifically a zinc-metallopeptidase enzyme with carboxypeptidase activity [111]. ACE2 participates in the production of the vasodilator angiotensin 1–7 by metabolizing angiotensin I (Ang I) and belongs to the renin-angiotensin system (RAS) involved in the dynamic control of vascular function such as vasoconstriction and blood pressure [112].

**Figure 4:**
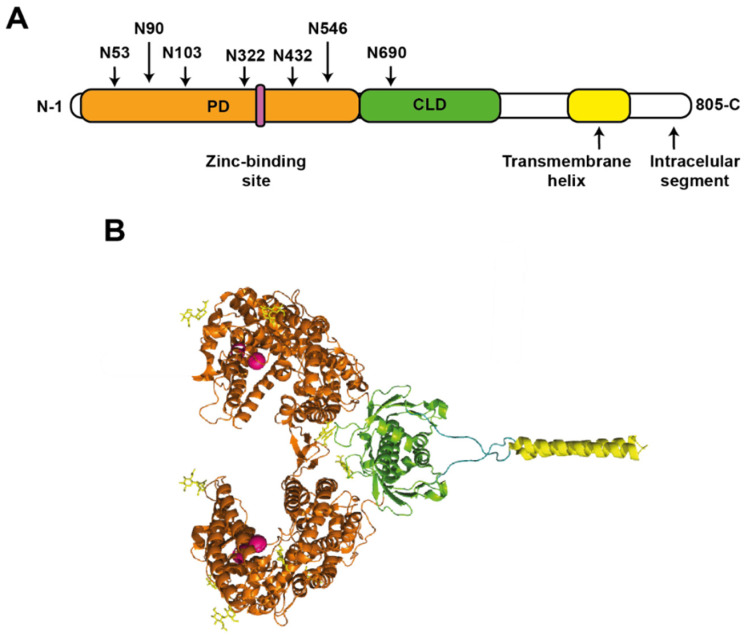
Angiotensin-converting enzyme 2 (ACE2) structure. (**A**) Schematic representation of the ACE2 protein. Peptidase domain (PD) and collectrin-like domain (CLD) are also shown. The N-glycosylation sites are shown as arrows for each amino acid residue, indicating their relative position in PD and CLD. (**B**) Structure of ACE2. According to panel A, the domains and motifs are shown, except the intracellular segment (PDB: 6M17).

ACE2 is 805 amino acids in size and has a molecular mass of 90–120 kDa in its glycosylated form [113,114]. In the N-terminal to C-terminal direction, it is composed of a peptidase domain (PD) (amino acids 19 to 615), which can be divided into subdomain I (amino acids 19–102, 290–397, and 417–430) and subdomain II (amino acids 103–289, 398–416, and 431–615) [115]. After PD, there is a collectrin-like domain (CLD) (amino acids 616 to 768), followed by a single transmembrane helix (amino acids 740–768), which binds to CLD through a long linker. Finally, an intracellular segment of ~ 40 amino acids is found at the C-terminal [116,117] (Figure 4A). Furthermore, ACE2 has a zinc-binding site coordinated by amino acids 374-HEMGH-378 belonging to a subdomain I (Figure 4B), a site that coordinates a chloride atom in subdomain II as well as six N-glycosylation sites in the PD (N53, N90, N103, N322, N432, and N546) [115,116,117,118]. ACE2 is available as a homodimer, stabilized by polar interactions between PD residues [116].

The location of ACE2 in the plasma cell membranes makes it a susceptible target for the recognition of viruses, having a critical role in the infection mechanism of the SARS-CoV-2 virus, which uses ACE2 as a receptor during the early infection process [2].

### 4.2. ACE2 of Animals and Cell Tropism

It is clear that the host range of SARS-CoV-2 is dictated by binding to the viral receptor; thus, the risk of infection in animals can be predicted in part by determining which ACE2 can form complexes with SARS-CoV-2 S glycoprotein [20]. Analysis of RBD from SARS-CoV-2 and ACE2 structures by simulation models have predicted the susceptibility to SARS-CoV-2 in the main wild and domestic mammalian species. The interaction of SARS-CoV-2 RBD and ACE2 through their structural affinity has been previously analyzed [119,120,121].

In one study, vertebrate ACE2 sequences were analyzed, and it was proposed that mammalian ACE2 has from medium to high probability of being used as a receptor for SARS-CoV-2 infection. This study was based on the sequences of ACE2 containing the 25 amino acids important for binding to the S glycoprotein from SARS-CoV-2. This study particularly suggested that ACE2 from catarrhine primates provides a high risk of SARS-CoV-2 infection. Thus, the chimpanzee, the green monkey, and the rhesus macaque may have a very high risk of infection. On the other hand, mammalians, such as killer whale and dolphin, may have just a high risk. The wild yak, squirrels, the golden hamster, cattle, the water buffalo, the goat, the cat, the rabbit, and the alpaca may have a medium risk. Instead, the panda, the camel, the rhino, the bear, the dog, the horse, the donkey, and the pig could pose a low risk. Finally, the guinea pig, the ferret, the sea lion, the Chinese pangolin, the house mouse, and the civet would have a very low risk of infection by SARS-CoV-2 [122]. There is also evidence that domestic cats, dogs, and mink can be positive for the SARS-CoV-2 antigen or antibodies against this virus [123]. Similarly, experimental inoculation of hamsters and cats with SARS-CoV-2 has demonstrated that these animal species are susceptible to this infection [124,125], whereas alpacas and pigs are not [126,127]. Under natural conditions, SARS-CoV-2-positive household dogs and cats generate antibodies to SARS-CoV-2, but PCR tests are usually negative [128]. Nevertheless, one study showed that infectious SARS-CoV-2 can be isolated from cats 5–6 days post-infection without symptoms, and these can infect other cats [125]. Besides, cats infected with SARS-CoV-2 can sometimes develop inflammation in the respiratory tract [124]. Fortunately, previously infected cats can elicit immune responses by preventing the transmission of SARS-CoV-2 to other cats [129]. On the other hand, ferrets can also be infected by SARS-CoV-2, but in these animals, this infection is poorly transmissible, whereas dogs do not produce a suitable infection and are less susceptible to this infection [124]. In the case of minks, 170 variants of SARS-CoV-2 were found. Several of these mutations were found in the S gene, which could compromise the effectiveness of the vaccines, although it would not affect the infectious or lethal capacity of the virus. The preventive measure of euthanasia was taken in the case of millions of minks [130].

It has also been demonstrated that SARS-CoV-2 uses ACE2 from civets and non-human primates as a viral receptor, but mice are resistant to SARS-CoV-2 infection. Nevertheless, in a mouse model of COVID-19, genetically modified mice expressing human ACE2 under the cytokeratin 18 (K18) promoter have been used. These mice develop severe lung disease after SARS-CoV-2 infection, resulting in 100% mortality when they are inoculated with 10^5^ PFU of SARS-CoV-2 [131]. Thus, the simple insertion of human ACE2 in mice by transgenesis increases their susceptibility to SARS-CoV-2 infection and favors the development of COVID-19-like disease. Moreover, the *ACE2* gene can have synonymous and nonsynonymous mutations, which are rare, but some of them could modify the susceptibility to human coronavirus infections [122,132].

On the other hand, it has been suggested that overexpression of ACE2 can be a risk factor for COVID-19 exacerbation due to the observation that the elderly population receives drug prescriptions that increase the expression of ACE2 and therefore has a higher risk of developing severe COVID-19. Moreover, this population is also at high risk of severe disease caused by MERS-CoV, whose receptor is dipeptidyl peptidase 4 instead of ACE2 [133].

Cell tropism depends on several cellular factors and not only on the S glycoprotein-ACE2 interaction. In fact, another receptor for the SARS-CoV-2, glycoprotein S has been identified. The transferrin receptor interacts with glycoprotein S to allow viral entry. Furthermore, a mouse model expressing human transferrin but not expressing human ACE2 has been used to assess SARS-CoV-2 infection, and these mice were shown to be infected by this route. As expected, the use of antibodies against the transferrin receptor produced an antiviral effect in this model [134]. SARS-CoV-2 viral replication also involves non-specific uptake mechanisms, such as the endocytic pathway, where SARS-CoV-2 S glycoprotein is primed by cathepsin-containing endosomal-lysosomal compartments. In addition, SARS-CoV-2 can be isolated using Huh7, human airway epithelial cells, or the VeroE6 cell line. The VeroE6/TMPRSS2 line has been genetically engineered to constitutively express TMPRSS2, resulting in increased susceptibility to SARS-CoV-2 infection. VeroE6/TMPRSS2 cells infected with SARS-CoV-2 develop a cytopathic effect consisting of cell rounding, syncytium formation, and detachment [135].

### 4.3. ACE2 Domains Involved in SARS-CoV-2 Infection

**Figure 5:**
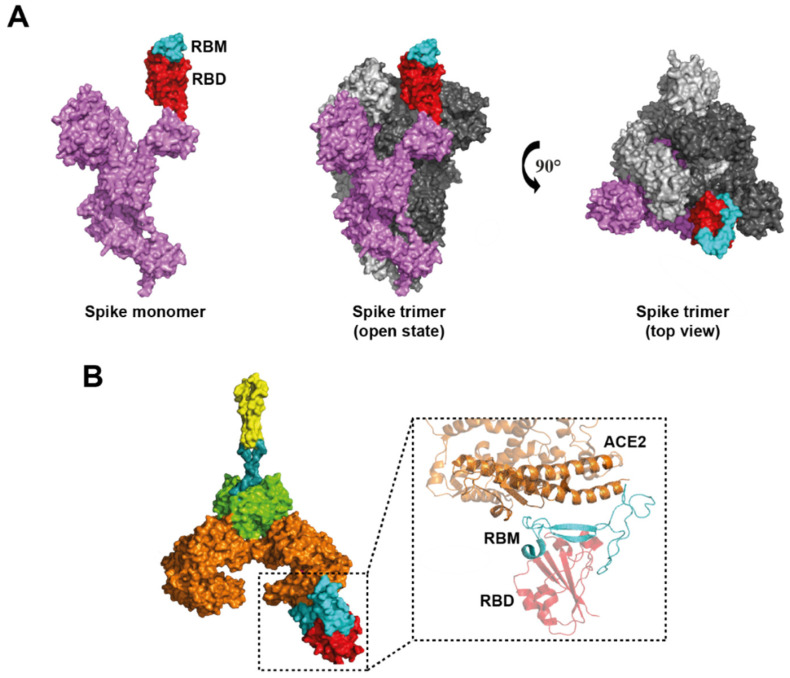
ACE2-RBD (S) complex. (**A**) Structure of a monomer of the S protein in lateral view of the open conformation and Spike trimer; also, the top view (PDB: 6VYB). The receptor binding domain (RBD) and receptor binding motif (RBM) are shown in red and cyan, respectively. (**B**) Structure of the ACE2-RBD complex, in which the amino acids of the RBM and the amino acids of the PD subdomain 1 of ACE2 interact (PDB: 6M17).

SARS-CoV-2 has a better affinity to recognize ACE2 than SARS-CoV-1, leading to the more rapid dissemination of SARS-CoV-2 [136,137]. Several research groups have solved the molecular structures of the SARS-CoV-2 glycoprotein S [137,138] (Figure 5A), ACE2 protein [115], and more recently the ACE2-RBD complex [116,138] (Figure 5B), which has allowed us to understand the infection mechanism of SARS-CoV-2 virions through glycoprotein S in addition to providing relevant information for the design and optimization of vaccines or therapies aimed at blocking ACE2 receptor binding [138]. S glycoprotein is composed of two subunits: subunit 1 (S1) that participates in ACE2 recognition through the receptor-binding motif (RBM), which is present in RBD; and subunit 2 (S2), which contains the elements for the fusion of the viral and host membranes [139]. The S protein is found as a homotrimer, either in a closed state or in a state open. In a closed state, all RBDs are hidden by the amino-terminal domain (NTD) of S glycoprotein. In a state open, one or more RBDs of the trimer are exposed to the surface and available to interact with ACE2 [140] (Figure 5A).

The ACE2-S complex is formed through the interaction of subdomain I of PD in ACE2 (primarily by residues of the N-terminal α-helix) and residues of the RBM present in RBD. It has been determined that 19 residues in ACE2 interact with 20 amino acids of RBD [138,139] (Figure 5B).

### 4.4. Is the ACE2 Variability Involved in COVID-19 Resistance?

The SARS-CoV-2 glycoprotein S has an affinity for the ACE2 receptor 10- to 15-fold higher than the SARS-CoV-1 glycoprotein S despite sharing a large percentage in its sequence [132]. That is why it was proposed that ACE2 polymorphisms could also contribute to susceptibility to SARS-CoV-2 infection, affecting the interaction of protein S with ACE2 and the COVID-19 severity.

Variability in the ACE2 amino acid sequence can have an effect on intra- and intermolecular interactions, affecting the ACE2-S interaction through changes between hydrogen bonds, π-π, and hydrophobic and hydrophilic interactions [141]. Other mutations may not cause such relevant conformational or interaction changes; however, they may cause changes in post-translational modifications of ACE2, as is the case with K26R, which is recognized for interfering in the coordination of a glycan of the critical N90 site to confer protection through glycan shielding, contributing to a phenotype more susceptible to infection [142].

**Figure 6:**
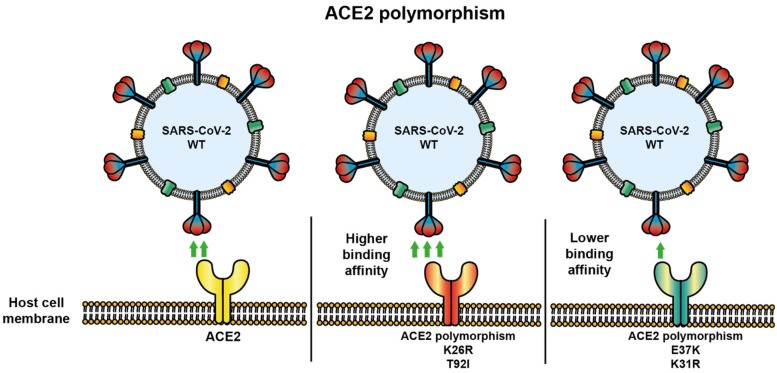
ACE2 polymorphisms modify their binding capability to SARS-CoV-2 glycoprotein S. ACE2 polymorphisms can also increase or decrease their binding affinity with SARS-CoV-2 glycoprotein S. Several ACE2 polymorphisms are shown interacting with SARS-CoV-2 wild type (WT). K26R and T29I ACE2 polymorphisms show a higher binding affinity to S glycoprotein. E37K and K31R ACE2 polymorphisms show a lower binding affinity to S glycoprotein, suggesting a lower infectivity to people with this last polymorphism.

In one prediction and structure study, ACE2 variants were analyzed according to the possibility of interaction with SARS-CoV-2 glycoprotein S. Variants with an increased susceptibility prediction were E23K, H378R, I21V, K26R, N64K, Q102P, S19P, T27A, and T92I, whereas variants with a possible protective effect against SARS-CoV-2 infection were D355N, D38V, D509Y E35K, E37K, F72V, G326E, G352V, H34R, K31R, K68E, M62V, N33I, N51S, Q388L, Y50F, and Y83H, showing decreased binding to SARS-CoV-2 glycoprotein S based on structural analysis. In addition, it was confirmed that K31R and E37K ACE2 polymorphisms had decreased affinity, whereas K26R and T92I ACE2 polymorphisms had increased affinity to S glycoprotein [132,142] (Figure 6).

Another important fact is that the mutation of amino acids M82, Y83, P84, and K353 in rat ACE2 converts this transmembrane protein into a receptor for SARS-CoV-1 [132,143]. Besides, the ACE2 viral receptor is encoding for a gene located in the X chromosome. Therefore, females express the *ACE2* gene as a mosaic pattern due to early X-inactivation. In contrast, males express a single ACE2 variant in all their cells, conferring the same susceptibility to COVID-19. ACE2 variants have been documented in humans, and their potential for SARS-CoV-2 binding may be affected. ACE2 sequencing in patients with extreme outcomes during SARS-CoV-2 infection, such as a death in infancy or asymptomatic super-spreaders, would be of great importance to characterize these rare variants of ACE2 [144]. Together, these results strongly suggest a protective effect in populations with specific ACE2 polymorphisms containing a low affinity to SARS-CoV-2 glycoprotein S, whereas other ACE2 polymorphisms could be highly susceptible to SARS-CoV-2 infection. This information can be used to perform risk analysis.

It has been shown that not only mutations that affect amino acid composition are capable of affecting ACE2 activity and its relationship with SARS-CoV-2. One study reported that a polymorphism with intronic localization was capable of modulating ACE2 levels in serum, possibly due to the increase in the strength of the splice site, resulting in a high expression, with a protective effect against SARS-CoV-2. Low levels of ACE2 in serum have been shown to contribute to severe SARS-CoV-2 infection [145], whereas high expression of ACE2 in serum may contribute to a protective effect against severe COVID-19 [146].

### 4.5. ACE2 Regulation during Inflammation

ACE2 modulates the RAS, vascular function, and blood pressure related to several signaling pathways in human cells. Because ACE2 is the receptor for coronaviruses, such as the human coronavirus HCoV-NL63, SARS-CoV-1, and SARS-CoV-2, the study of the regulation of its expression has been suggested as central and necessary [147].

As ACE2, along with TMPRSS2 and exopeptidase CD26 (also known as DPP4), have critical roles in cell entry and viral infectivity, it has been proposed that global DNA methylation, including *ACE2* gene methylation and histone modifications, may lead to different levels of susceptibility to viral infections, such as COVID-19 [148]. Some studies have evaluated the epigenetic implications of ACE2 expression. For example, in one study, nearly 700 lung transcriptomes from COVID-19 patients with comorbidities were analyzed. The results obtained for COVID-19 patients were compared with those from healthy individuals. A high expression of *ACE2* and genes associated with epigenetic modulation (including *HAT1*, *HDAC2*, and *KDM5B*) as well as potential genes for the SARS-CoV-2 infection, such as RAB1A, were detected in these patients. Therefore, patients with comorbidities have overexpression of the viral receptor ACE2 and other proteins that participate in the SARS-CoV-2 infection, and this has been associated to COVID-19 severity [149].

On the other hand, ACE2 expression increases with age, which is associated with inflammatory responses [11]. A bioinformatics study showed that the ACE2 and *IL-6* gene promoters are activated by inflammatory and interferon signaling. During severe COVID-19, the cytokine storm is characterized mainly by the overexpression of IL-6. IL-6 and ACE2 overexpression is caused by tumor necrosis factor (TNF)-α, TLR, and IFN signaling. In this scenario, histones modifications and chromatin remodeling facilitate binding of transcription factors, such as nuclear factor κB (NFκB) and interferon regulatory factor (IRF), on the promoter of IL-6 and ACE2. In this way, the SARS-CoV-2 infection can regulate the expression of IL-6 and ACE2, increasing their expression through the reactivation of inflammation [12]. Thus, the epigenetic changes associated with IL-6 and ACE2 could be used as biomarkers to predict susceptibility to severe COVID-19 in different groups of people.

Furthermore, obese patients suffer from a chronic inflammatory state and have a large number of ACE2 receptors located on adipocytes. COVID-19 causes severe systemic inflammation and decreased HO-1. Changes in the clinical course of the disease are given by the interaction of three polymorphisms: first, the CYP2D6 enzyme system; second, the anti-inflammatory gene *HO-1*; and third, the enzyme system’s ACE2 [150].

## 5. Other Polymorphisms of Transmembrane Proteins, Cell Surface Molecules, or Enzymes Involved in COVID-19

### 5.1. TMPRSS2 and DC26 Polymorphisms

After SARS-CoV-2 binds to ACE2, S glycoprotein is processed by TMPRSS2. Mainly, TMPRSS2 is co-expressed with ACE2 in: type 2 pneumocytes, enterocytes, and nasal goblet secretory cells. ACE2 mutations are more important than TMPRSS2 mutations [20]. Nevertheless, both TMPRSS2 and exopeptidase CD26 are also membrane-bound proteins involved in SARS-CoV-2 infection. The genetic variants of these proteins were analyzed in a global study of 26 populations. Several variants of TMPRSS2 (rs112657409, rs11910678, rs77675406, rs713400, and rs430915) were identified [151,152], and it has been proposed that single nucleotide-polymorphisms from TMPRSS2 and its non-coding RNA-dependent regulation may be responsible for different levels of susceptibility to SARS-CoV-2 among several populations [153]. Moreover, epigenetic modification at the rs13015258-C allele (a 50 UTR variant from CD26) may increase CD26 expression and the susceptibility to COVID-19 in patients with type 2 diabetes. The epigenetic modifications implicated in the susceptibility to COVID-19 will be briefly discussed later [151].

### 5.2. Tolloid Like-1 Polymorphism

Another enzyme involved in the COVID-19 development is Tolloid-likeprotein 1 (TLL1). Particularly, the A allele of TLL1 rs17047200 is found in the blood and has been associated with a more severe disease. The A allele of TLL1 rs17047200 has also been found in the liver with fibrosis using a murine model of hepatocellular carcinoma. It was proposed that this SNP could modify the splicing of TLL1 mRNA, generating short variants that have high catalytic activity, prompting hepatic fibrogenesis [154,155]. Interestingly, rs17047200 SNP also correlates with COVID-19 severity. The AA genotype of rs17047200 had a higher predisposition to develop COVID-19, as genotyping analysis indicated [19].

### 5.3. Cathepsin B and L

Cathepsin B and L are cysteine proteases found in the endosomal compartment, and they prime SARS-CoV-2 S glycoprotein for viral entry. The variant rs10831496 of cathepsin C has been associated with severe COVID-19 [152].

### 5.4. ABO Polymorphism

The *ABO* gene responsible for determining blood type has been suggested to be associated with the severity of COVID-19. However, in one study, it was suggested the ABO rs912805253 variant as a risk factor for SARS-CoV-2 infection rather than the risk of hospitalization or death from COVID-19 [156]. Nevertheless, other studies have not been able to confirm this association [157].

## 6. Polymorphisms in Immune System Molecules

### 6.1. Cytokine Polymorphism

Cytokines are crucial regulators of the immune response against infections. Thus, cytokines can prompt inflammation, their resolution, immune cell activation, cytotoxic T lymphocyte (CTL) responses, antibody responses, tissue damage, and tissue repair. IL-1β, IL-8, IL-18, and TNF-α are related to inflammation and tissue damage, whereas IL-10 is related to immune tolerance and antibody response. IL-4 is also related to antibody response and is produced by CD4+ T cells, mainly by T-helper cell type II (Th2 immune response). On the other hand, IFN-γ can activate macrophages and is related to immune responses against intracellular pathogens where CD8+ T cells are assisted by a Th1 immune response [99]. Activation of macrophages, epithelial cells, and endothelial cells release pro-inflammatory cytokines, such as IL-1β, and IL-18, which cause neutrophilia and leukopenia, thus leading to tissue damage and insufficient immune response [158,159]. High levels of cytokines, such as IL-6, IL-1β, TNF-α, and IFN-γ, have been detected in COVID-19 patients, causing a syndrome called cytokine storm, which is believed to be the main cause of tissue damage in the pathophysiology of COVID-19 [160]. Moreover, more than 80% of COVID patients have lymphopenia, and critically ill patients develop acute respiratory distress syndrome, requiring mechanical ventilation [161].

On the other hand, macrophages and T lymphocytes secrete IL-8 and IL-17 to recruit primarily neutrophils in response to a viral infection. Under normal conditions, neutrophils undergo apoptosis, but this process can be inhibited under inflammatory states characterized by the presence of IL-8. Interestingly, during infection by SARS-CoV-2, there are alterations in the innate response, which explains the low expression of IL-8 due to a decrease in the chemoattraction ability and survival of neutrophils [162]. The IL-10 rs1800896 polymorphism was positively correlated with COVID-19 prevalence, whereas the IL-17 rs2275913 polymorphism was negatively correlated with mortality rate due to this disease [163].

Cytokine polymorphisms can affect the transcription level or the sequence of cytokine genes. Consequently, the level of expression and/or its function is affected. The immune system is involved in the cytokine storm that generates adult respiratory distress syndrome [19]. According to the cases described in the United States, well-established polymorphisms have been found in alleles that encode cytokines such as TNF-α, INF-α/β, IL-4 and cytokine receptors, including IFN-α and IFN-β receptor subunit 2 (IFNAR2) and interleukin 1 receptor antagonist (IL-1RA encoded by *IL-1RN*). According to world mapping, an IFNAR2 polymorphism has been found with high frequency in Latin America and Europe, whereas IFN-β polymorphisms have been found in Africa and Asia, and something similar is observed for *IL1RN*. The different expression of cytokines caused by point mutations in these genes interferes in the evolution of the immune response and in the different clinical outcomes of COVID-19. These polymorphisms can also affect the response to the use of therapies and vaccines [164].

Type III IFNs are also important during viral infections. IFN-λ is a type III IFN, and its function is similar to type I IFNs because it has antiviral properties. Similarly, type I IFN and type III IFN activate the same signaling pathway, although they have different receptors [165,166]. The binding of the IFN-λ to its receptor (IFN-λ receptor or IFNLR) generates activation of the STAT signaling pathway through phosphorylation [167]. Unlike type I IFNs, IFN-λ receptors are expressed on epithelial barrier surfaces, including the blood-brain barrier, gastrointestinal tract, and respiratory epithelial tissue. IFN-λ polymorphisms have been associated with protection against viral diseases [165]. Besides, simple nucleotide polymorphisms (SNPs) associated with IFN-λ signaling have also been associated with treatment outcomes in patients with hepatitis B and C virus infection [167]. Particularly, COVID-19 patients who contain the CC genotype of IFN-λ rs12979860 have a more major incidence of COVID-19 than individuals without the polymorphism. Moreover, patients containing the alleles C and A have a poor COVID-19 outcome [19].

### 6.2. Polymorphisms in Tyrosine Kinases

Cytokine (and growth factors) signaling is given by the Janus kinase (JAK)/STAT pathway [168]. The tyrosine kinase 2 (*TYK2*) belongs to the Janus kinase family, and this gene is found on chromosome 19p13.2 [14]. Near the *TYK2* gene has been found the rs2109069 polymorphism in the dipeptidyl peptidase 9 (*DPP9*) gene (chromosome 19p13.3). DPP9 is a protease that cleaves C-X-C motif chemokine ligand 10 (CXCL10), an antiviral molecule. Additionally, DPP9 is involved in inflammation and antigen presentation, whereas TYK2 is overexpressed during the development of severe diseases [14,169].

Another innate immune polymorphism related to the development of COVID-19 is discoidin domain receptor 1 (DDR1) rs4618569. DDR1 is a tyrosine kinase receptor activated by collagen and involved in cytokine production, cell differentiation, and the modulation of adhesion molecules [170]. Patients containing DDR1 rs4618569 had high C reactive protein (CRP), D-dimer, ferritin, high risk of mechanical ventilation, as well as severe COVID-19 and an increase in the mortality rate [19]. All these results together support the suggestions that polymorphisms of innate immunity are very closely related to the development of severe COVID-19 by the inadequate immune response after SARS-CoV-2 infection.

### 6.3. Chemokine Polymorphisms

Chemokines and their receptors also play an important role in severe COVID-19. COVID-19 patients show increased production of C-C chemokine receptor (CCR) 1, CCR2, and CCR5 in thoracic dorsal root ganglion neurons [171,172]. Interestingly, inflammatory macrophages from severe COVID-19 patients overexpress chemokine ligands (CCLs): CCL3, CCL20, CXCL1, CXCL3, and CXCL10. These chemokines have been found together with pro-inflammatory cytokines. Similarly, high levels of IL-6 and CCL5 (RANTES) as well as viremia and decreased CD8+ T cells are found in terminal COVID-19 patients. Because of this, it was proposed that the administration of CCR blockers could be used in patients with similar conditions. Later, it was demonstrated that the treatment of leronlimab antibody (CCR-5 blocker) restores the levels of CD4+ and CD8+ T cells, reduces plasmatic IL-6, and decreases the SARS-CoV-2 viremia [173]. Thus, it has been demonstrated that the inhibition of CCR pathways inhibits the exacerbated immune response.

The *CCR5* gene is located in chromosome 3p21, and a variant of this gene has been reported to have a 32 bp deletion, resulting in a truncated protein of 215 aa instead of 352 aa. This variant was named CCR5 Δ32 [174,175]. In one study, CCR5 Δ32 was positively correlated to SARS-CoV-2 infection and mortality rate. CCR5 and its ligand CCL5 have been shown to play an important role in the inflammatory response, most commonly by recruiting leukocytes to eliminate infectious pathogens [171,175].

Another gene involved is C-X-C motif chemokine receptor 6 (*CXCR6*), whose function is to allow homing of CD8+ T cells in the lungs, as occurs during infection by the influenza virus. In a SNP study, an association of six genes on chromosome 3p21.31 was found in COVID-19 patients with respiratory failure. These genes include *CCR9* and *CXCR6*. Italians and Spanish had a polymorphism in CXCR6, known as rs11385942, that contains an insertion-deletion of GA/A and was related to a decrease in CXCR6 and overexpression of solute carrier family 6 member 20 (SLC6A20) and leucine zipper transcription factor-like 1 (LZTFL1) in the lungs of COVID-19 patients that required mechanical ventilation [176].

Chemokine production is an important antiviral response associated with infiltration of immune cells in the infected lungs as part of the immune response against coronavirus. Although chemokines are vital in attracting the immune system and eliminating the virus, their overexpression can prompt the increase of inflammation and consequently adult respiratory distress syndrome, a common complication in COVID-19. Examination of lung from these patients revealed the expression of CCR2, the CCL2 receptor, CCL7, and CCL12 [177].

### 6.4. Polymorphisms in TLR and Their Signaling Molecules

TLRs are PRRs located on the cell surface or in endosomes from immune and non-immune cells (such as glia and neurons) [178]. In viral infections, TLRs located in endosomes are the most important PRRs. Overall, there are 11 human TLRs, which sense damage molecular patterns (DAMPs) and pathogen-associated molecular patterns (PAMPs). More particularly, TLR-7 and TLR-8 recognize single-stranded RNA (ssRNA), while intermediate RNA (double-stranded RNA or dsRNA) produced during viral replication is detected by TLR-3 [179,180,181]. TLR signal transduction implicates myeloid differentiation factor 88 (MyD88) and TIR domain-containing adaptor-inducing interferon-beta (TRIF), activating NF-kB, type I IFN regulatory factor (IRF)3, and IRF7, leading to a type I IFN response [16,182].

TLR SNPs have been found in neoplastic, autoimmune, and infectious diseases. For instance, rs3775291 polymorphism of TLR-3 was associated with autoimmune diseases, including rheumatoid arthritis, systemic lupus erythematosus, and sarcoidosis [16]. In the case of SARS-CoV-2 infection, rs3775291 (L412F) and rs3775290 polymorphisms have caused recognition of SARS-CoV-2 RNA to be decreased [14,183]. Actually, the rs3775291 polymorphism has been related to susceptibility to infectious diseases, including SARS-CoV-2 infection, and now, it is a marker for severity and mortality in COVID-19. Thus, TLR-3 deficiency is associated with high susceptibility to RNA virus infection, and the deficiency or mutation of the rs3775291 polymorphism prevents its expression and is also associated with diabetes and pulmonary hypertension. This leads to a rapid progression of COVID-19 in infected patients [15,184].

Recent studies have identified deleterious variants involved in IFN type I signaling, including variants of TLR-3; transcription factors, such as STAT1 and STAT2; interferon regulatory factors, such as IRF1 and IRF7; and IFN-α receptors, such as IFNAR1 e IFNAR2, are associated with inadequate immune responses after vaccination as well as the most severe cases of COVID-19. This is reflected at the cellular level, where pDCs cannot produce IFNs during SARS-CoV-2 infections [16,185]. In another work, TLR-3, IRF3, and IRF7 variants were detected in patients with severe COVID-19. The TLR-3 variants were found to be associated with impaired immunity to SARS-CoV-2. T cells from a patient harboring an IRF7 deficient variant had low levels of IRF7, and the pDCs from this patient did not produce type I or III IFNs when exposed to SARS-CoV-2. Similarly, T cells from another patient with IFN-α/β receptor 1 (IFNAR1) deficiency (IFNAR1 p.Pro335del) had an affected IFN-α2/IFN-β response. Furthermore, fibroblasts (transduced with ACE2 and TMPRSS2) containing this mutation had a more marked SARS-CoV-2 infection than cells from healthy donors. In the same work, patients harboring TICAM1 or TBK1 deficiency were tested to evaluate IFN-α levels during the acute phase of COVID-19. These patients had very low levels of IFN-α (<1pg per ml) [17]. Very similar results have been observed in severe COVID-19 patients containing auto-antibodies directed against IFN, but these IFN levels may vary [17,186,187]. Similarly, the TLR-3 p.Pro554Ser variant [188] as well as the IRF3 and IRF7 variants with loss-of-function have also been detected in patients with severe pneumonia by influenza virus [17,189,190]. Although it seems logical that when the mechanisms involved in IFN production are damaged, individuals may be more likely to develop severe COVID-19, other work found no association between the variants of the genes involved in IRF7- and TLR-3-dependent type I IFN pathway reported by Zhang et al. in 2020. However, in this work, the associations between the STAT2 variant 12-56744928-GA and the TLR-3 variant 12-56744928-GA with severe COVID-19 and mild COVID-19, respectively, were detected [18]. This could indicate that Zhang’s results are not a rule of thumb and that the genetics of the host involved in the development of severe COVID-19 is highly complex.

It has been strongly suggested that TLR-4 can also be associated to severe COVID-19. TLR-4 recognizes lipopolysaccharide but can also recognize structures from viruses and even mycoplasma and fungi [178]. TLR-4 is able to interact with SARS-CoV-2 S glycoprotein or its epitopes through hydrophobic interactions (as indicated by predictive analysis) [178,191,192,193], and this added to its neuroinvasive capability leads to the overactivation of TLR-4. The TLR-4-Spike complex generates an aggravated inflammation accompanied by TNF-α, IL-1β, and IL-6 [178]. This elevated level of pro-inflammatory cytokines prompts TLR-4 overexpression [193]. The uncontrollable release of these cytokines causes cytokine storm that can generate acute respiratory distress syndrome and multiple organ failure [194]. In addition, the interaction between SARS-CoV-2 S glycoprotein and TLR-4 also causes overexpression of IFN-stimulated genes, including *ACE2*, which facilitates the viral entry [178,195].

It should be noted that TLR-4 plays an important role against respiratory coronavirus. A natural mutation of the *TLR-4* gene has been reported to alter its function. A strain of mice was genetically modified to contain this mutation. C3H/HeJ mice containing this mutation were infected with mouse hepatitis virus strain 1 (MHV-1), and their mortality and morbidity rates were compared with wild-type C3H/HeN mice. The results indicated that C3H/HeJ mice harboring the TLR-4 mutation, despite improving airway function, had higher morbidity and mortality rates than wild-type mice [196]. Similar results have been observed in humans containing TLR-4 polymorphisms after respiratory syncytial virus infection. The TLR-4 D259G and T359I polymorphisms were investigated in children who developed a severe respiratory syncytial virus (RSV) infection. Interestingly, children with TLR-4 mutations were associated with severe RSV infection. In the same study, the association of CD14 polymorphisms and severe RSV infection were investigated, but no predisposition to this infection was found [196,197].

TLR-7 variants have also been associated with severe COVID-19 (c.2129_2132del; p.(Gln710Argfs*180 and c.2383G>T; p.(Val795Phe)) [139,197], and this can be explained as a defect in the detection of viral RNA by this receptor with loss-of-function [152,198]. TLR-8 variants could also be related to SARS-CoV-2 infection and COVID-19.

Other RNA sensors implicated in the development of severe COVID-19 could be retinoic acid-inducible gene I (RIG-I) and MDA5, which detect viral RNA, promoting type-I IFN production. *RIG-I* is an IFN-stimulated gene. Its activation leads to signal transduction that activates the NFκB, IRF3, and JAK-STAT pathways. It is probable that SARS-CoV-2 is also detected by these cytosolic RNA sensors, and it could be suggested that loss-of-sense mutations in these sensors or the proteins associated with their transduction signaling can also cause poor viral clearance, as observed with other coronaviruses [16,199,200].

### 6.5. HLA Polymorphism

Human leukocyte antigen (HLA) molecules are important immune regulatory components encoded by MHC genes. The HLA complex constitutes a specific group of molecules expressed on the cell surface; these are crucial for recognizing peptides (epitopes) by the adaptive immune system. These epitopes derived from pathogens are charged on the surface of antigen-presenting cells (APCs) and presented to T lymphocytes, triggering the cellular immune response. HLA genes exhibit extreme diversity and have several thousand reported polymorphisms. The *HLA* gene is located in the short arm of human chromosome 6 (6p21.3). Genetic differences in HLA genes account for individual variations in the immune response against pathogens. HLA polymorphisms are implicated in susceptibility to infectious diseases, such as hepatitis B (HBV), hepatitis C (HCV), chikungunya, dengue, and influenza A (H1N1) viruses. Hence, the HLA system is highly polymorphic and is organized as class I and II. HLA class I contains A, B, and C, whereas class II encloses DR, DP, and DQ. Generally, these HLA classes are related to antigen presentation to T lymphocytes and the recognition of proteins. Some studies about HLA alleles in COVID-19 patients have recently demonstrated their association with disease severity and progression [201,202].

A small observational and prospective study to determine HLA polymorphisms was performed in 72 patients diagnosed with COVID-19 and 3886 healthy individuals. Ten of the 72 patients with COVID-19 were non-survivors. Determination of HLA genetic polymorphisms showed that the alleles HLA-A*11, HLA-C*01, and HLA-DQB1*04 could be associated with mortality at 30 days for patients with COVID-19 [203]. In another study with 82 patients with COVID-19, a significantly higher frequency of the HLA-C*07:29 and HLA-B*15:27 alleles were found in patients with COVID-19 when compared with healthy individuals [204]. The in-silico analysis of candidate peptides based on comparison with immunogenic peptides resulted in the prediction of SARS-CoV-2-associated peptides. These peptides could bind to various HLA alleles (both class I and class II), including HLA-A:02:01, implicated in the activation of effector T cells [205].

HLA class I polymorphisms, such as HLA-B*46:01, HLA-B*07:03, and HLA-Cw*08:01, and HLA class II polymorphisms HLA-DRB4*01 and HLA-DRB1*12:02 have been associated with the predisposition to COVID-19. Notably, the HLA-B*46:01 allele was found to bind to the fewest SARS-CoV-2 peptides. Therefore, it is a non-protective allele that could increase susceptibility to COVID-19 [206]. This HLA-B*46:01 allele is mainly found in South East Asia and is absent in India and Africa, with a low distribution in European populations [207,208]. In contrast, HLA-B*15:03 seems to be broadly protective since it has a high ability to present peptides of SARS-CoV-2 and other coronaviruses. Thus, HLA-B*46:01 may be a susceptibility marker for SARS-CoV-2 infection, whereas the HLA-B*15:03 allele protects against this infection [209].

Furthermore, a protective effect for HLA-DRB1*03:01, HLA-Cw*15:02, and HLA-A*02:01 has been proposed. HLA-A*24:02 was associated with COVID-19 susceptibility after it was detected in a small sample size composed of four of five patients from Wuhan [209]. The HLA allele frequency distribution analysis in a group of 99 Italian patients showed a significant association of HLA-DRB1*15:01 and HLA-DQB1*06:02 with susceptibility to COVID-19 [210]. The decreased expression of HLA-DR molecules on circulating monocytes (mHLA-DR) altered inflammatory cytokine release profile and increased lymphopenia prompt immunosuppression. Patients with SARS-CoV-2 show decreased mHLA-DR expression [211] (Table 2).

### 6.6. Polymorphisms in Other Immune Proteins

Three proteins implicated in immune response or inflammation have been identified as possible candidates related to severity to COVID-19. These genes include *CADM1*, *ZBTB16*, and *REXO2*. The genes that code for these proteins are found near rs1712779, but more studies are needed to clarify their association with severe COVID-19 [152]

Non-classical HLA class I molecules with tolerogenic activity, such as HLA-G and HLA-E, have also been associated with COVID-19. HLA-G comprises membrane-bound isoforms and soluble forms. In addition, a soluble isoform is generated from membrane-bound HLA-G by matrix metalloproteinases (MMPs). Six receptors recognize HLA-G; these include immunoglobulin-like transcript (ILT)-2, ILT-4, killer cell immunoglobulin-like receptor 2DL4 (KIR2DL4), CD8, CD160, and NKG2A/CD94. HLA-G molecules suppress the immune system by inhibiting NK cell, CD8+ T cells, CD4+ T cells, and dendritic cell function [215,216]. HLA-G expression on the surface of infected cells can be enhanced by viruses favoring replication and spreading without immune reactions from the host. A high expression of HLA-G in the cell membrane was reported in the early inflammation stage in Chinese patients with COVID-19. Disease progression and its complications seem to be related to a high expression of HLA-G on the surface of infected cells, whereas a reduction in HLA-G expression in immune cells, such as T cells, B cells, and monocytes, was observed in COVID-19 patients [217].

The HLA-E molecule has been suggested to be highly expressed in infected cells from COVID-19 patients [218]. HLA-E can interact with its receptors to regulate immune cell functions. NKG2C (encoded by *KLRC2* gene) activates an NK cell receptor that binds to HLA-E on infected cells, activating NK cells. HLA-E*0101/0103 genetic variants are caused by a single nucleotide polymorphism. The deletion of KLRC2 and HLA-E*0101/0103 allelic variants were evaluated in a study cohort of 361 patients, of which 92 had mild COVID symptoms, and 269 patients had severe COVID-19. The results indicated that *KLRC2* deletion is a significant individual risk factor for severe COVID-19. In addition, the HLA-E*0101 allele was associated with complications requiring hospitalization and intensive care. NKG2C+ NK cell-mediated immune responses may be critical in SARS-CoV-2 infections. These immune responses can be affected by genetic variants in the NKG2C/HLA-E axis [219].

During the development of COVID-19, epigenetic modulation is also important. Significant differences in the genome-scale DNA methylation (DNAm) profiles of peripheral blood mononuclear cells (PBMCs) were identified from nine critical COVID-19 patients compared with the controls. This DNAm signature was characterized by (1) the hypermethylation of IFN-related genes and (2) the hypomethylation of inflammatory genes. The epigenetic clock (named GrimAge) strongly suggests that the risk of severe COVID-19 and its mortality rate are associated with the enhancement of DNAm. This blood-based epigenetic clock may be useful for providing information about the association between epigenetic modulation and susceptibility to SAR-CoV-2 infection and its progression and outcome [220].

## 7. Discussion

In this work, we analyzed viral and host genetic factors related to the susceptibility to the immunopathogenesis of COVID-19. It is well-known that the virulence and infectious capacity of viral diseases are given by mutations in viral proteins [221,222] and host genetics [206,207,208,209]. RNA viruses, including coronaviruses, have a higher mutation rate than DNA viruses, jumping the species barrier [70,223]. For instance, the S1 domain of the S protein facilitates the interaction between SARS-CoV-1 and its receptor, ACE2. Rats are not susceptible to SARS-CoV-1 infection, but a single amino acid mutation in this domain during viral adaptation to rats through serial passages increased its affinity to ACE2 in this rodent [224]. Similarly, a recombinant SARS-CoV-2 containing mutations Q498Y/P499T in the S gene (SARS-CoV-2 MA) was developed by molecular modeling and reverse genetics by modifying SARS-CoV-2 RBD to enable its interaction with ACE2 from mice. Interestingly, mice infected with SARS-CoV-2 MA developed hemorrhage in the lung and inflammation at four days post-infection [225]. Thus, two simple mutations of amino acids of SARS-CoV-2 develop a COVID-19-like disease in a mice model.

On the other hand, the host genetic factors are also important during SARS-CoV-2 infection. For SARS-CoV-2 infection, the first step is binding of S glycoprotein to ACE2 receptor from host cells. It has been observed that the distribution of ACE2 receptor is different in populations. For instance, the expression of ACE2 in podocytes (kidney cells) of Occidentals is higher than in Asians [13]. Besides, several studies have strongly suggested that some ACE polymorphisms could induce susceptibility to COVID-19, whereas others are resistant to this disease due to their low binding ability with SARS-CoV-2 glycoprotein S, as demonstrated by affinity experiments [132] (Figure 6). In addition, membrane-bound proteins TMPRSS2 and exopeptidase CD26 are also implicated in SARS-CoV-2 infection. It has been demonstrated that the polymorphism of these proteins can also increase the susceptibility to COVID-19 [151,153].

The suitable immune response against SARS-CoV-2 is also related to resistance to infection. At the beginning of SARS-CoV-2 infection, no antibodies against this virus were present in the individuals [226,227]. Therefore, it is clear that the innate immune response plays a pivotal role at the beginning of SARS-CoV-2 infection, where recognition of viral structures, such as RNA and glycoprotein S, must be accurately detected by PRRs (mainly TLR-3 and TLR-4) found on the plasma membrane and endosomes of APCs. Here, the genetic background may dictate the outcome of COVID-19 patients due to some polymorphisms of these PRRs, cytokines, or molecules involved in their signaling being associated with increased risk, while others are associated with protection.

Fortunately, after infection, most infected people are asymptomatic or develop mild symptoms of COVID-19 [95,228,229]. Interestingly, these people generate SARS-CoV-2-specific T cells, whereas people with severe COVID-19 contain low levels of T cells [230]. For T-cell activation, APCs play a pivotal role because they are responsible for antigen uptake, its processing, and presentation of immunogenic peptides through MHC molecules. The MHC is naturally polymorphic in the population. It is well known that some polymorphisms of MHC molecules are associated with higher susceptibility to some diseases. Such diseases include rheumatoid arthritis [231], psoriasis, systemic lupus erythematosus, ankylosing spondylitis, multiple sclerosis, type 1 diabetes, and Crohn’s disease, among others [232]. Additionally, it has been demonstrated that some MHC polymorphisms can also be responsible for susceptibility to viral infections, including HBV, HCV, human immunodeficiency virus, human papillomavirus, and dengue, as genome-wide association studies (GWAS) have indicated [232,233]. Similarly, protection against influenza depends on MCHII, as demonstrated in experiments with transgenic mice. In this case, mice expressing DRB1*0401 (susceptible to auto-immunity) and *0402 mice were immunized with H1N1 and challenged with H3N2 influenza virus. Only *0401 mice produced a cross-protective immune response against H3N2 influenza strain. The MHCII molecules of these mice had a traffic toward late endosome/lysosomes, whereas MHCII molecules of *0402 mice moved toward early lysosomes [234], indicating that although some MHC polymorphisms are associated with autoimmune diseases, the same MHC molecules can play a pivotal role in the clearance of some viral infections. In the case of SARS-CoV-2 infection, HLA-A*11, HLA-C*01, and HLA-DQB1*04 are associated with mortality by COVID-19 [203], whereas another HLA, such as HLA-A:02:01, is implicated in the activation of T cells [205]. Similarly, HLA class I molecules, such as HLAB*46:01, HLA-B*07:03, and HLA-Cw*08:01, as well as HLA class II molecules, including HLA-DRB4*01 and HLA-DRB1*12:02, are associated to predisposition to COVID-19, and some of these HLA molecules have been shown to bind to few SARS-CoV-2 peptides [206]. This suggests that the genetic predisposition to severe COVID-19 can be owing to inadequate bind of SARS-CoV-2 peptides to HLA molecules in APCs and therefore activation of a low number of T-helper cells and cytotoxic T cells.

Novel RNA vaccines against SARS-CoV-2 are generating a robust, long-lasting immune response. The Pfizer-BioNtech vaccine is one of the most protective vaccines against COVID-19. However, its administration appears to induce a variable immune response in the population. Vaccine effectiveness was calculated to 95% for Danish people [235] although it also depends on the age since only a 64% effectiveness in people 84 years old and a 90% effectiveness in health workers has been observed [161]. Thus, genetic susceptibility due to different immune polymorphisms partly could explain the variability of protection after vaccination in different continents.

Nowadays, several SARS-CoV-2 variants continue emerging, and these will need to be monitored. SARS-CoV-2 omicron variant is one of the newest, and it has at least 50 mutations identified, with 32 of those mutations involving the S glycoprotein. When compared to the reference strain, the SARS-CoV-2 omicron variant, many of the most concerning mutations occur in the RBD. This has suggested that its susceptibility to immune protection elicited by the existing COVID-19 infection and vaccines may be altered. Very little is known about either infectivity or transmissibility at this time. The available data from clinicians at the front lines in South Africa suggest that patients with the omicron variant are younger people with a clinical presentation similar to that of past variants. However, information should be treated with caution given that severe COVID-19 cases typically present several weeks after the initial symptoms associated with mild disease [236,237].

## 8. Concluding Remarks

Susceptibility to severe COVID-19 is a complex and poorly understood phenomenon. In the present study, we addressed current research about the genetic variability of SARS-CoV-2 and the genetic susceptibility to severe COVID-19. The identification of variants from SARS-CoV-2 that increase its rate of infection should be epidemiologically monitored and adequately contained to avoid its dissemination and generation of new variants, which may be even more infectious and virulent. Several genetic factors predispose people to severe COVID-19. The main factors involve the ACE2 polymorphism. Prediction, structure analysis, experimental affinity, and clinical studies of patients with severe COVID-19 have indicated that some ACE2 polymorphisms have a higher affinity for protein S. During SARS-CoV-2 infection, severe inflammation favors the expression of ACE2 and TLR-4. Some polymorphisms of TLRs, chemokines, cytokines, and their receptors are associated with severe COVID-19. Another polymorphic factor implicated with genetic susceptibility to SARS-CoV-2 infection and the severity of COVID-19 is the HLA polymorphism, which has direct implications with the immune system, including the activation of T-helper and cytotoxic T cells through the presentation by mature APCs. In addition, epigenetic changes in the ACE2 receptor and in cytokine genes, such as *IL-6*, are also associated with severe COVID-19. Finally, identification of host genetic and epigenetic factors that increase susceptibility to SARS-CoV-2 infection can predict the severity of COVID-19 and aid in the early treatment and prognosis in these patients.

## Figures and Tables

**Table 1:** Mutations in S glycoprotein of SARS-CoV-2 variants.

SARS-CoV-2 Variants	Spike Mutations
B.1.1.7	69-HV-70 del, 144-Y del, N501Y, A570D, D614G, P681H, T716I, S982A, and D1118H
B.1.351	L18F, D80A, D215A, 242-LAL-244 del, R246I, K417N, E484K, N501Y, D614G, and A701V
P.1	L18F, T20N, P26S, D138Y, R190S, K417T, E484K, N501Y, D614G, H655Y, and T1027I
B.1.617 and sub-lineages	T19R, T95I, G142D, E154K, 156-157 del, R158G, L452R, T478K, E484Q, D614G, P681R, D950N, and Q1071H

**Table 2:** Polymorphisms implicated in the development or resistance to COVID-19 and their relationship with the immune system.

Gene	Implications in COVID-19	Susceptibility Polymorphisms	Resistance Polymorphisms
*TMPRSS2*	A protease that cleaves glycoprotein S for its priming leading to virus entry.	rs112657409, rs11910678, rs77675406, and rs713400 variants can regulate the expression of TMPRSS2 and could be implicated in SARS-CoV-2 infection [151]. rs430915 allele A has been associated with overexpression of TMPRSS2 in lungs [152].	No data reported.
*CTSB* (encoding for cathepsin C)	Cysteine protease priming glycoprotein S for viral entry.	rs10831496 is associated to severe COVID-19 [152].	No data reported
*KANSL1*	A nuclear protein involved in histone acetylation.	No data reported.	Variant rs1819040:T>A was associated with protection [156].
*ABO*	Responsible for determining blood type.	rs912805253 variant was suggested as a risk factor for SARS-CoV-2 infection [156].	No data reported.
*ACE2*	The SARS-CoV-2 receptor: mediates viral attachment and membrane fusion. Under inflammation, ACE2 is overexpressed. Now, ACE2 is considered an IFN-stimulated gene.	E23K, H378R, I21V, K26R, N64K, Q102P, S19P, T27A, and T92I. K26R and T92I have increased affinity to SARS-CoV-2 glycoprotein S [132].	D355N, D38V, D509Y E35K, E37K, F72V, G326E, G352V, H34R, K31R, K68E, M62V, N33I, N51S, Q388L Y50F, and Y83H. K31R and E37K have decreased affinity to SARS-CoV-2 S glycoprotein [132].
*FOXP4*	A forkhead transcription factor that regulates the specific transcription activity in cells.	Variant rs1886814:A>C is associated with the development of COVID-19 and interstitial lung disease [156].	No data reported.
*IL-10*	Related to immune tolerance and antibody response.	rs1800896 [163].	No data reported.
*IL-17*	Recruits neutrophils in response to a viral infection.	No data reported.	rs2275913 [163].
*IFN-λ*	It has antiviral properties and can prompt the expression of IFN-stimulated genes.	rs12979860 (CC genotype) as well as C and A alleles [19].	No data reported.
*TLL1*	A metalloprotease implicated in the morphogenesis of the heart. This enzyme can also activate SARS-CoV-2 S glycoprotein.	rs17047200 (AA genotype) [19,212].	No data reported.
*DPP9*	A protease that cleaves CXCL10, an antiviral molecule. It is involved in inflammation and antigen presentation.	rs2109069 and rs12610495 are related to critical illness and interstitial lung disease, respectively [14,156,169].	No data reported.
*DDR1*	A tyrosine kinase receptor activated by collagen and involved in cytokine production, cell differentiation, and the modulation of adhesion molecules.	rs4618569 [19].	No data reported.
*CCR5*	CCR5 and its ligand CCL5 play an important role in the inflammatory response by recruiting leukocytes to eliminate infectious pathogens.	CCR5 Δ32 [171,175].	No data reported.
*CXCR6*	Allows homing of CD8+ T cells in the lungs.	rs11385942 [176].	No data reported.
*TYK2*	A member of Janus kinases protein families. It is associated with cytoplasmic domains of cytokine receptors prompting their signaling though phosphorylation.	rs74956615:T>A variant confers risk for COVID-19, whereas the missense variant rs34536443:G>C (also p.Pro1104Ala) has been correlated with risk of hospitalization (but it is protective against autoimmune diseases) [156].	No data reported.
*TLR-3*	Detects intermediate dsRNA during viral replication.	rs3775291 and rs3775290 [14,15,183,185]. Other variants are p.Ser339fs, p.Pro554Ser, p.Trp769*, and p.Met870Val [17]. In another work, the TLR-3 variant 12-56744928-GA was associated with mild COVID-19, while no association was found with the variants mentioned above [18].	No data reported.
*TLR-7*	Detects ssRNA from viruses prompting IFN production.	4 young male patients were reported to have developed severe COVID-19. These patients were identified with loss-of-function variants of TLR-7, including a 4-nucleotide deletion (c.2129_2132del; p.(Gln710Argfs*18)) and a missense variant (c.2383G>T; p.(Val795Phe)) [152,198].	No data reported.
*IRF3*	As its name implies, it is an interferon regulatory transcription factor (IRF). IRF3 includes phosphorylation sites at its C-terminal, a DNA-binding domain, and a nuclear localization signal.	p.Glu49del and p.Asn146Lys variants [17].	No data reported.
*IRF7*	Interacts with IRF3, and together, they regulate the IFN-α genes.	p.Pro364fs/p.Pro364fs, p.Met371Val/p.Asp117Asn, p.Arg7fs, p.Gln185*, p.Pro246fs, p.Arg369Gln, and p.Phe95Ser variants [17].	No data reported.
*IFNAR1/IFNAR2*	A receptor found in the cell membrane, and it contains both IFNAR1 and IFNAR2.	p.Trp73Cys/Trp73Cys, p.Ser422Arg/Ser422Arg, and p.Pro335del variant from IFNAR1 as well as p.Glu140fs variant from IFNAR2 [17].	No data reported.
*TICAM1*	Also known as TLR adaptor molecule 1. Its function is to mediate the interaction between TLR-3 and signal transduction proteins activating NFκB.	p.Thr4Ile, p.Ser60Cys, and p.Gln392Lys variants [17]. Other TICAM1 variants have been related to pneumonia in Chinese children [213].	No data reported.
*TBK1*	It is a protein kinase that phosphorylates IRF3, causing its nuclear translocation to induce the transcription of type-1 IFN genes.	p.Phe24Ser and p.Arg308* [17]. TBK1 mutations are also found in children with encephalitis caused by herpesvirus infection [214].	No data reported.
*STAT2*	Signal transducer and activator of transcription 2 is associated with IRF9. Upon phosphorylation, STAT2 forms a multimeric complex, which binds to a specific DNA sequence to activate type-1 IFN genes.	STAT2 variant 12-56744928-GA has been associated with severe COVID-19 [18].	No data reported.
HLA class I	A protein used for binding to processed peptides after antigen processing. HLA class I bound to SARS-CoV-2 epitopes to stimulate anti-SARS-CoV-2 CD8+ cells prompting lysis of infected cells.	HLA-A*11 HLA-C*01 HLA-C*07:29 HLA-B*15:27 HLA-B*46:01 HLA-B*07:03 HLA-Cw*08:01 HLA-B*46:01 HLA-A*24:02 [203,204,206,209]	HLA-B*15:03 HLA-Cw*15:02 HLA-A*02:01 [209]
HLA class II	Presents epitopes to CD4+ lymphocytes to enhance the cytotoxic effect of CD8+ T lymphocytes (Th1) or enhance antibody production (Th2). Some HLA polymorphisms have low binding capacity, predisposing to COVID-19.	HLA-DQB1*04 HLA-DRB4*01 HLA-DRB1*12:02 HLA-DRB1*15:01 HLA-DQB1*06:02 [203,206,210]	HLA-DRB1*03:01 [209]
